# A novel anesthesia regime enables neurofunctional studies and imaging genetics across mouse strains

**DOI:** 10.1038/srep24523

**Published:** 2016-04-15

**Authors:** Marija M. Petrinovic, Georges Hankov, Aileen Schroeter, Andreas Bruns, Markus Rudin, Markus von Kienlin, Basil Künnecke, Thomas Mueggler

**Affiliations:** 1 Roche Pharmaceutical Research & Early Development, Neuroscience Discovery, Roche Innovation Center Basel, F. Hoffmann-La Roche Ltd, Grenzacherstrasse 124, 4070 Basel, Switzerland; 2 Institute for Biomedical Engineering, University and ETH Zurich, Wolfgang-Pauli-Strasse 27, 8093 Zurich, Switzerland; 3 Neuroscience Center Zurich, University and ETH Zurich, Winterthurerstrasse 190, 8057 Zurich, Switzerland; 4 Institute of Pharmacology and Toxicology, University of Zurich, Winterthurerstrasse 190, 8057 Zurich, Switzerland

## Abstract

Functional magnetic resonance imaging (fMRI) has revolutionized neuroscience by opening a unique window that allows neurocircuitry function and pathological alterations to be probed non-invasively across brain disorders. Here we report a novel sustainable anesthesia procedure for small animal neuroimaging that overcomes shortcomings of anesthetics commonly used in rodent fMRI. The significantly improved preservation of cerebrovascular dynamics enhances sensitivity to neural activity changes for which it serves as a proxy in fMRI readouts. Excellent cross-species/strain applicability provides coherence among preclinical findings and is expected to improve translation to clinical fMRI investigations. The novel anesthesia procedure based on the GABAergic anesthetic etomidate was extensively validated in fMRI studies conducted in a range of genetically engineered rodent models of autism and strains commonly used for transgenic manipulations. Etomidate proved effective, yielded long-term stable physiology with basal cerebral blood flow of ~0.5 ml/g/min and full recovery. Cerebrovascular responsiveness of up to 180% was maintained as demonstrated with perfusion- and BOLD-based fMRI upon hypercapnic, pharmacological and sensory stimulation. Hence, etomidate lends itself as an anesthetic-of-choice for translational neuroimaging studies across rodent models of brain disorders.

Functional magnetic resonance imaging (fMRI) has evolved into one of the principal translational methodologies for investigating central nervous system (CNS) function and treatment effects of pharmacological interventions in various neurological and neuropsychiatric disorders^1^,^2^. The non-invasive nature and translatability of fMRI allows for repeated assessments in the same subject and cross-species investigations. However, these genuine advantages of fMRI have not yet been fully leveraged for studies in murine models of human CNS pathologies. In contrast to studies in man, small animal imaging generally resorts to anesthesia to keep animals lying still during examinations. Controlled anesthetic conditions are also advantageous over the use of awake animals, as they minimize stress-related neurofunctional and peripheral changes that may introduce confounds and image artefacts even after extensive acclimation training^3^.

Current fMRI modalities are based on readouts of local cerebrovascular and hemodynamic responses to changes in cerebral oxygen and energy consumption that are taken as proxy for neural activity. Anesthesia introduces potential confounds as it has differential effects on local cerebral function and may compromise this neurovascular coupling. The choice of the optimal anesthesia regime for fMRI is further complicated by the fact that different species and even different strains may react differently to the same anesthetic^4^. Moreover, for fMRI reporting on sensory paradigms, the anesthetic should have a minimal analgesic component, whereas for pharmacological fMRI (phMRI), the interaction with the receptor system targeted by the pharmacological intervention to be investigated should be avoided or minimized^5^,^6^.

To date, α-chloralose, isoflurane and medetomidine are widely used anesthetics for fMRI studies in rats and mice^3^,^7^. Among these, α-chloralose shows the least depressant effects on brain activity and neurovascular coupling, but is unsuitable for longitudinal fMRI studies due to its toxicity^8^. The volatile anesthetic isoflurane excels with ease of use and rapid reversibility of its anesthetic effect but causes neural, cardiovascular and respiratory depression^9^,^10^,^11^,^12^. Notably, it also raises basal cerebral blood flow (CBF) in a dose-dependent manner^11^ that potentially limits neurovascular reactivity and thus would leave only a narrow window for hemodynamic imaging readouts. At lower doses of isoflurane, muscle relaxants and artificial ventilation may be required^13^,^14^,^15^, which results in a more demanding experimental setup particularly in mice. Medetomidine, an α_2_-adrenoceptor agonist, has been introduced to fMRI as an alternative to α-chloralose and has proven successful in few commonly used wild-type rat and mouse lines^15^,^16^,^17^,^18^. However, major species and strain differences have been found (as also reported below) for medetomidine efficacy, even including resilience to sedation^19^. With the advent of an increasing number of genetically engineered mouse models of CNS disorders the need has emerged for anesthesia protocols that can be reliably applied across various rodent strains and thus allow cross-comparative fMRI studies to be carried out. Therefore, we explored the potential of etomidate, a rapidly acting anesthetic with GABA_A_ receptor subtype specific mode-of-action and low analgesic properties^20^. Etomidate is preferentially used in critically ill patients as it provides superior hemodynamic stability compared to other anesthetic agents^21^. In spontaneously breathing mice, etomidate was reported to maintain arterial blood pressure within the physiological range^22^. Also, basal CBF values ascertained by different modalities were found to be substantially lower compared to isoflurane and cerebrovascular autoregulation was preserved^22^,^23^. This pharmacological profile putatively renders etomidate particularly suitable for fMRI studies in small rodents.

Here, we report on studies in which we used continuous arterial spin labelling (CASL)-based fMRI to quantitatively assess and benchmark local brain perfusion, cerebrovascular reserve capacity (CVRC) and functional responses to pharmacological intervention in freely breathing mice and rats anesthetized with either isoflurane, medetomidine or according to our newly introduced protocol based on etomidate. We show that etomidate-based anesthesia overcomes the shortcomings of anesthetics commonly used in rodent fMRI related to compromised hemodynamic responses and limited cross-species/strain applicability, and we report findings in six different mouse models of autism spectrum disorder. Etomidate anesthesia provides long-term stable physiological conditions and full recovery, hence lending itself as an anesthesia-of-choice for comparative and repeated fMRI-based endophenotyping and imaging genetics studies across rodent models.

## Results

### Isoflurane abolishes CVRC in mice

**Figure 1:**
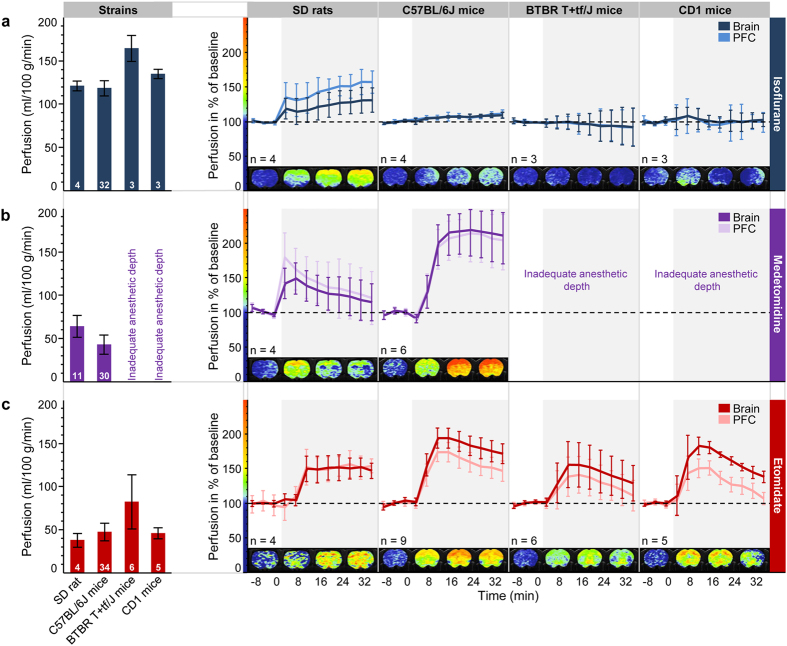
Comparison of cerebral perfusion at rest and upon injection of acetazolamide in spontaneously breathing SD rats, C57BL/6J, BTBR T+tf/J and CD1 mice under different anesthetic conditions. Left panels: mean cerebral perfusion at rest under (**a**) isoflurane, (**b**) medetomidine or (**c**) etomidate anesthesia. Numbers in the bars reflect sample sizes, error bars indicate standard deviations. Right panels: time course of perfusion response in whole brain and the PFC before and after injection of acetazolamide as a proxy for cerebrovascular reserve capacity. Responses are presented as percentage change relative to baseline (black dashed line at 100%) to allow comparison of different regions of interest. The left border of the gray-shaded area designates the time-point of acetazolamide application. Acetazolamide (30 mg/kg) was injected intravenously in isoflurane- and medetomidine-anesthetized animals and intraperitoneally under etomidate, thus explaining the slightly delayed response onset for the latter. Data are shown as sample means, and error bars represent 95% confidence intervals to visualize significant deviations from baseline. Sample sizes are provided below each graph. Color-coded magnetic resonance images represent the percentage change of perfusion (according to the color bar along the y axis) as an average over three consecutive time-points for a representative coronal plane (rats: +1.00 mm; mice: +1.34 mm relative to bregma). PFC, prefrontal cortex; SD, Sprague Dawley.

Isoflurane at a maintenance dose of ~1.1-fold the minimal alveolar concentration (rats: 2.0–2.3%; mice: 1.8–2.0%) required to attain stable anesthesia in non-paralyzed, spontaneously breathing rodents^24^ resulted in robustly elevated basal perfusion as shown in Fig. 1a (left panel). Whole-brain perfusion assessed by CASL-based fMRI was at 120 ml/100 g/min in Sprague-Dawley (SD) rats and at strain-specific 120–165 ml/100 g/min in C57BL/6J, CD1 and BTBR T+tf/J mice, respectively (ascending order). These values further document the well-known vasodilatory effect of isoflurane on cerebral vasculature^25^. Shallow isoflurane anesthesia in combination with neuromuscular blockade and artificial ventilation was shown to keep CBF within a lower range and fMRI reported neurovascular responses upon sensory stimuli^13^,^14^,^15^. Under the present conditions in spontaneously breathing animals, CVRC may be exhausted and cerebrovascular coupling be abrogated if resistance vessels are maximally dilated. CVRC was therefore explored by injection of the carbonic anhydrase blocker acetazolamide, a well-established vasodilatory stimulus that increases brain perfusion^26^. Figure 1a (right panel) demonstrates in rats that despite a high basal brain perfusion, isoflurane anesthesia afforded sufficiently large a window for neurofunctional vascular responses, as reported earlier^27^. Notably in cortical regions, which feature high basal perfusion, a strong response was observed. Analogous settings in mice however completely abolished CVRC and resulted in the absence of changes in brain perfusion (Fig. 1a, right panels).

### Medetomidine fails to reliably sedate different mouse strains

Medetomidine-based anesthesia in SD rats and C57BL/6J mice resulted in brain perfusion within the range reported earlier by radiotracer and MRI methods^12^,^22^ (Fig. 1b, left panel). In both species, perfusion was found to be significantly lower (*p* < 10^−15^) than the elevated levels observed for isoflurane anesthesia. Notably, under medetomidine CVRC was preserved in SD rats as well as in C57BL/6J mice, as demonstrated by a marked acetazolamide-induced increase in perfusion across the brain and specifically also in the cortex (Fig. 1b, right panels). In mice, the average peak responses (see Methods) were significantly higher (*p* < 10^−7^ both for brain and cortex) than the negligible effects under isoflurane. Medetomidine failed, however, to reliably sedate BTBR T+tf/J and CD1 mice even at substantially increased doses. This finding is in alignment with previous reports on the influence of genetic backgrounds on the hypnotic susceptibility to anesthetics^4^. The limited effectiveness of medetomidine likely reflects species- and strain-related differences in ligand affinity to and density of the α_2_- adrenoceptors.

### Novel etomidate-based anesthesia regime for fMRI

For introducing etomidate-based anesthesia for fMRI, we first established dosing conditions that are suitable for reliable and sustained anesthesia in spontaneously breathing mice of different strains and allow full recovery (see Methods and Table 1). Etomidate was delivered as an intravenous infusion into a lateral tail vein. In brief, mice of all strains tested received a priming dose of 4 mg/kg/min over 3 min followed by a continuous infusion of etomidate that was adjusted depending on the genetic background (C57BL/6J: 0.75 mg/kg; BTBR T+tf/J: 1.0 mg/kg; CD1: 1.5 mg/kg) such as to achieve a constant and comparable anesthetic depth. SD rats could be reliably anesthetized by priming with 5 mg/kg/min of etomidate, followed by constant infusion of 0.25 mg/kg/min. Breathing rate, righting reflex, sensory and nociceptive responses were used as indicators of anesthetic depth and recovery following anesthesia. Animals anesthetized for 1 h recovered and were fully ambulatory ~1 h post anesthesia. Etomidate’s negligible analgesic properties often resulted in mild reactions to tactile stimuli. Therefore, for induction of anesthesia and subsequent positioning of the animals in the MRI scanner, supportive isoflurane anesthesia was applied during the initial 8 min. Thereafter, isoflurane was suspended whereas the delivery of the carrier gas (oxygen-enriched air) was continued throughout the MRI assessments.

### Etomidate preserves CVRC in mice and rats

In a second step, we investigated etomidate’s effects on physiology and particularly on cerebral perfusion and CVRC. Respiratory rate of the spontaneously breathing mice was stable at 90–130 breaths/min and thus remained within the physiological span reported for resting animals (see Supplementary Fig. S1). Body temperature could also be readily kept within very narrow limits close to the target of 37.3 °C (see Supplementary Fig. S1 online). Notably, in all mouse and rat strains investigated total brain perfusion was within the range ascertained previously (40–80 ml/100 g/min)^12^,^22^ (Fig. 1c, left panel). Baseline perfusion in rats and mice was found to be significantly lower (BTBR T+tf/J mice: *p* < 0.01; others: *p* < 10^−7^) than the elevated levels under isoflurane anesthesia. Most relevantly, however, CVRC was preserved as demonstrated by profound increases in perfusion both in total brain and locally in prefrontal cortex (PFC) that were elicited upon acetazolamide injection (Fig. 1c, right panels). In rats, the effects were of the same order of magnitude as under isoflurane (significant difference only for whole brain: *p* = 0.01). For mice, average peak responses were significantly higher than the negligible effects under isoflurane (BTBR T+tf/J mice: *p* < 0.01; other mouse strains: *p* < 10^–5^, both for brain and PFC). Remarkably, in C57BL/6J mice etomidate had very similar effects on perfusion and CVRC to those seen for medetomidine (Fig. 1b). For further substantiation of the CVRC observed, saline was injected in place of acetazolamide as a negative control. As expected, saline prompted only minor changes in brain perfusion, regardless of the anesthetic used (see Supplementary Fig. S2 online).

### Etomidate enables imaging genetics across mouse lines

**Figure 2:**
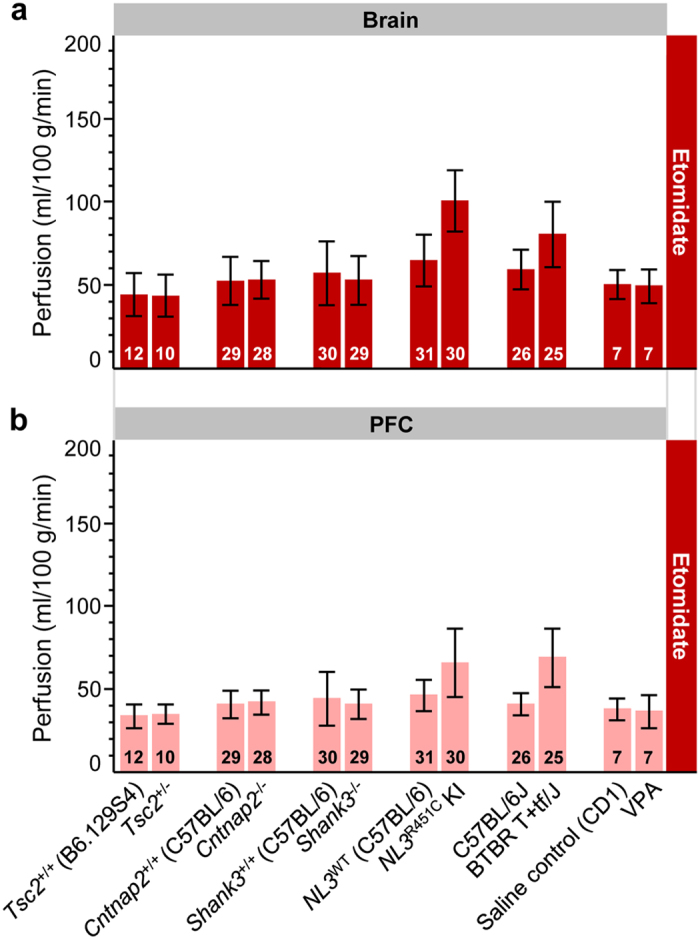
Comparison of basal blood perfusion in the brain and PFC of six mouse models of autism spectrum disorder under etomidate anesthesia. Perfusion in the (**a**) brain and (**b**) PFC was assessed in spontaneously breathing *Tsc2*^+/−^, *Cntnap2*^−/−^, *Shank*3^−/−^, *NL3*^R451C^ KI, BTBR T+tf/J, VPA-exposed mice, and corresponding wild-type controls. Sample sizes are provided in each bar. Error bars indicate standard deviations to visualize the spread of data for individuals. All the models were on a C57BL/6J background, except VPA (CD1) and *Tsc2*^+/−^ (B6.129S4 mixed background). Wild-type littermates were used as controls, except for BTBR T+tf/J mice where age-matched C57BL/6J mice served as controls. KI, knock-in; PFC, prefrontal cortex; VPA, valproic acid; WT, wild-type.

In light of the importance and increasing number of rodent models of human CNS disorders, we have assessed brain perfusion in six mouse models of autism spectrum disorder and respective wild-type controls. These models encompassed tuberous sclerosis *Tsc2*^+/−^ mice as genetic model of syndromic autism^28^, *Cntnap2*^−/−^, *Shank3*^−/−^ and *NL3*^R451C^ knock-in (KI) mice for idiopathic autism^29^,^30^,^31^, the valproic acid (VPA) mouse model of environmentally triggered autism^32^, as well as BTBR T+tf/J mice as a behaviorally defined model of autism of unknown genetic origin^33^. Under etomidate anesthesia, perfusion at rest in total brain and PFC was highly consistent among models and respective wild-type littermates except for BTBR T+tf/J (age-matched C57BL/6J mice were used as controls) and *NL3*^R451C^ KI mice which exhibited increased perfusion as compared to their controls (Fig. 2a,b). Notably, the latter increases were larger than the typical variations of cerebral perfusion across the different cohorts of wild-type C57BL/6J, CD1 and B6.129S4 mixed background animals. Taken together, these findings attest high reproducibility and demonstrate the suitability of etomidate-based anesthesia for imaging genetics studies by reporting neurofunctional consequences of genetic mutations.

### Etomidate supports pharmacological fMRI

**Figure 3:**
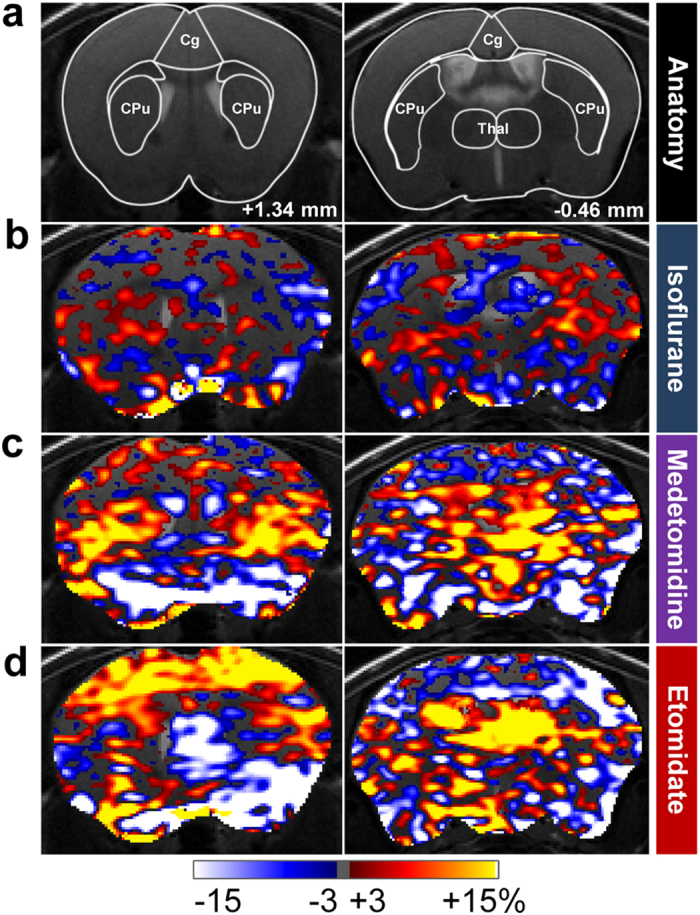
Pharmacological fMRI upon acute challenge with the antipsychotic olanzapine in C57BL/6J mice under different anesthetics. (**a**) Mouse brain atlas superimposed on T_2_-weighted anatomical images with outlined regions of interest and indicated distance to bregma. (**b–d**) phMRI activation maps showing differences in normalized perfusion between olanzapine- (3 mg/kg) and vehicle-treated C57BL/6J mice anesthetized with either (**b**) isoflurane, (**c**) medetomidine or (**d**) etomidate. Differences are given in percent, according to the color bar. Sample sizes for vehicle- and olanzapine-treated animals, respectively, were n = 8 and 9 under isoflurane, n = 8 and 8 under medetomidine, and n = 6 and 8 under etomidate. Cg, cingulate cortex; CPu, striatum; Thal, thalamus.

We further validated the etomidate-based anesthesia regime for its application to fMRI studies of neuropharmacological modulations. Here, CASL-based phMRI was employed to exemplarily investigate the neurofunctional effects of the second generation antipsychotic olanzapine in C57BL/6J mice anesthetized with isoflurane, medetomidine and etomidate, respectively (Fig. 3a–d). In line with the CVRC data, acute dosing with olanzapine (3 mg/kg, intraperitoneally) in C57BL/6J mice elicited distinct region-specific changes (versus vehicle-treated controls) under medetomidine and etomidate sedation (Fig. 3c,d). Under isoflurane, however, no major pharmacological response was observed with phMRI (Fig. 3b), which is in line with the restricted CVRC reported above for this condition. Specifically, the average (i.e., root-mean-square) response strengths across all 42 brain areas delineated in the in-house digital atlas (see Methods) were 2.0% (95% confidence interval: 1.7…2.6%), 6.7% (5.9…8.8%) and 11.2% (9.6…14.5%) for isoflurane, medetomidine and etomidate, respectively. The underlying perfusion changes were observed in key regions known to be engaged by antipsychotic treatment in rodents such as the striatum^34^, cortex and also thalamus^35^.

### Etomidate is compatible with sensory fMRI

**Figure 4:**
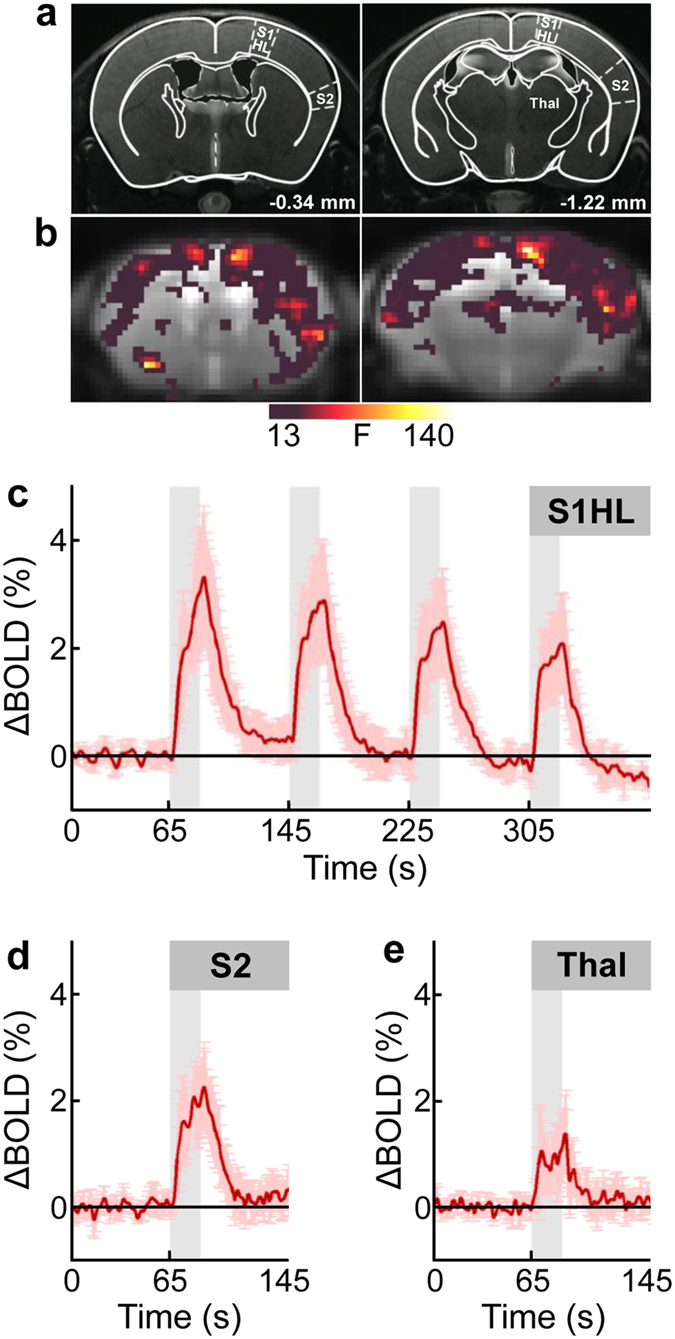
BOLD fMRI of neural activation elicited by unilateral electrical hindpaw stimulation in etomidate-anesthetized C57BL/6J mice. (**a**) Mouse brain atlas superimposed on T_2_-weighted anatomical images with distances to bregma indicated. Regions of interest are outlined in the hemisphere contralateral to the stimulated hindpaw. (**b**) Statistical parametric maps of two brain sections, located at −0.34 mm and −1.22 mm relative to bregma, showing neural activation pattern upon electrical stimulation (2 mA, 5 Hz). The strongest stimulus-induced activation was observed both in ipsi- and contralateral hemisphere S1HL and S2 cortices, and Thal. The color bar represents F-values (F = 13 corresponds to *p* < 0.05, family-wise error rate (FWER) corrected with minimum cluster size ≥20 voxels). (**c**) Time course of BOLD signal change (∆BOLD; in percent of baseline values) in contralateral S1HL during the four stimulation periods of 20 s duration each (gray bars). (**d,e**) ∆BOLD (shown for the first stimulus period only) in contralateral (**d**) S2 and (**e**) Thal illustrating region-specific activation strength. Data are depicted as mean ± standard deviation for n = 5 animals. BOLD, blood oxygenation level dependent; S1HL, primary somatosensory hindlimb cortex; S2, secondary somatosensory cortex; Thal, thalamus.

The scope of etomidate-based anesthesia in fMRI was further established by investigating the hemodynamic response to sensory stimuli based on the commonly used electrical hindpaw stimulation paradigm. Corresponding with previous investigations performed in rodents^13^,^14^,^15^, in this study mice were mechanically ventilated and paralyzed using a muscle relaxant. Neurofunctional responses were assessed by blood oxygenation level dependent (BOLD) fMRI. Unilateral hindpaw stimulation at 2 mA in etomidate-anesthetized C57BL/6J mice reproducibly evoked BOLD activation in both the ipsi- and contra-lateral primary and secondary somatosensory hindlimb cortices, and thalamic areas (Fig. 4a–e). These areas are known to be involved in somatosensory and nociceptive processing, and their activation is in line with previous findings in isoflurane- and medetomidine-anesthetized mice^15^,^16^.

## Discussion

Preserved neurovascular coupling and ensuing hemodynamic responses to neural activity are essential prerequisites for current fMRI modalities, which have become invaluable tools for assessing neurofunction in rodent models of human brain disorders. Particularly in mice, anesthetics commonly used for fMRI are either ineffective in particular strains^4^ or tamper with the neurovascular response^9^. Here, we have established and validated a novel anesthesia regime based on etomidate that addresses these shortcomings and enables neurofunctional endophenotyping and imaging genetics studies across different mouse strains and lines using sensory and pharmacological paradigms.

Etomidate has been reported to slightly reduce cerebral blood flow^36^. Notably, in our study we observed absolute perfusion values under etomidate that were comparable to and in C57BL/6J mice even slightly higher than those under medetomidine. This set point of cerebral perfusion is distinctly lower than under isoflurane yet well within the previously reported range for basal perfusion^12^,^22^. Hence, it affords an enlarged cerebrovascular reserve capacity (as demonstrated here upon acetazolamide challenges) that is required to translate responses to neural activity into high-fidelity neurovascular readouts.

Disparate functional effects observed under different anesthesia regimes point to a very fundamental theme in functional and pharmacological MRI, i.e., the putative interaction between drug, paradigm or strain of the animals on the one hand and the anesthetic’s mode-of-action^37^ on the other hand. For resting state fMRI in C57BL/6J mice the combined use of isoflurane and medetomidine has recently been proposed as suitable anesthesia based on phenomenological characteristics of the observed functional connectivity patterns^38^. However, action of medetomidine is strongly strain-dependent and thus is a shortcoming of particular importance in view of the ever growing number of genetically engineered mouse models of human CNS disorders - many of which are not based on the C57BL/6J genetic background. In our phMRI studies in mice under medetomidine and etomidate anesthesia, the specific spatial response patterns observed for olanzapine may in part reflect interactions between olanzapine’s antagonism at the dopamine D_2_ and serotonin 5-HT_2A_ receptors with the anesthetics’ α_2_-adrenergic and GABAergic modes-of-action, respectively^20^,^39^. The weak striatal response to olanzapine under etomidate may suggest an interaction between olanzapine’s effect on local dopamine receptors and etomidate-mediated alterations of GABA currents via the GABA_A_ receptor β3-subunit expressed in striatal medium spiny neurons^40^. In cortical area, on the other hand, the well-described pronounced reduction in norepinephrine release prompted by medetomidine via presynaptic adrenergic α_2_ auto-receptors interferes with the olanzapine-mediated alteration of prefrontal cortex activity, thus explaining the differential drug response observed under medetomidine and etomidate in this brain region. The choice of anesthetic for neuro-functional MRI investigations remains one of the most critical decisions to be taken in order to avoid undue interactions. Etomidate with its distinct high specificity for GABA_A_ receptor β2/3-subunits represents in this respect an attractive option for investigating drug effects which may else be blocked or masked by broader modes-of-action of other anesthetics.

Etomidate provides narcotic levels adequate for reliable fMRI assessments over several hours, long-term stable physiological conditions and foremost preserves neurovascular coupling and CVRC in spontaneously breathing animals. Etomidate also affords full recovery from anesthesia and is suitable for repeated and longitudinal studies. Prolonged etomidate anesthesia has been reported to transiently suppress adrenal steroidogenesis in a dose-dependent and reversible fashion^41^. The clinical relevance of these effects, however, has remained controversial^21^. We neither observed any adverse effects on animals’ health and behavior, nor needed to adjust doses even after prolonged and multiple dosing for repeated fMRI assessments. With the current efforts in developing etomidate analogues that are more rapidly metabolized^41^, the putative metabolic liability of etomidate may be readily addressed. Commercially available formulations of etomidate are provided for intravenous infusion only. In small rodents, this adds substantial complexity to the experimental setup and thus novel formulations of etomidate suitable for subcutaneous or intraperitoneal infusion would be highly desirable.

In summary, we have demonstrated that etomidate-based anesthesia is suitable for obtaining high-fidelity neurovascular readouts in spontaneously breathing mice of different strains and lines as well as in rats. CASL-based phMRI and BOLD fMRI in rodents anesthetized with etomidate proved sensitive to pharmacological and sensory paradigms for modulation of neural activity, respectively. Hence etomidate lends itself as an anesthetic-of-choice for comparative and repeated fMRI across rodent models of brain disorders.

## Methods

### Animals

Ethical approval for this study was provided by the Federal Food Safety and Veterinary Office of Switzerland. All animal experiments were conducted in strict adherence to the Swiss federal ordinance on animal protection and welfare as well as according to the rules of the Association for Assessment and Accreditation of Laboratory Animal Care International (AAALAC), and with the explicit approval of the local veterinary authorities. The data presented in this manuscript were compiled from several studies carried out over a period of approximately five years. CASL imaging is particularly suitable for such cross-study analyses as it yields a calibrated quantity in physical units and provides a consistent and long-term stable readout^27^. In an effort to minimize animal suffering and to reduce the number of animals required reference data from relevant study arms of our in-house investigational phMRI studies were used to complement the dedicated studies.

Experiments were conducted on 31 adult (12–16 weeks old) male Sprague–Dawley (SD) rats (Charles River Laboratories, L’Arbresle, France), 5 adult (12–16 weeks old) female C57BL/6J mice (Janvier, Le Genest-St Isle, France), 38 adult (12–16 weeks old) male BTBR T+tf/J mice (The Jackson Laboratory, Bar Harbor, ME, USA), 16 adult (12–16 weeks old) male CD1 mice (Harlan Laboratories Inc., Boxmeer, The Netherlands), 28 adult (12–16 weeks old) *Cntnap2*^−/−^ mice and 29 *Cntnap2*^+/+^ littermates (The Jackson Laboratory, Bar Harbor, ME, USA; C57BL/6J genetic background), 29 adult (12–16 weeks old) *Shank3*^−/−^ mice and 30 *Shank3*^+/+^ littermates (The Jackson Laboratory, Bar Harbor, ME, USA; C57BL/6J genetic background), 30 adult (12–16 weeks old) *NL3*^R451C^ knock-in (KI) mice and 31 *NL3*^WT^ littermates (The Rockefeller University, NY, USA; C57BL/6J genetic background), 10 adult (12–16 weeks old) *Tsc2*^+/−^ mice and 12 *Tsc2*^+/+^ littermates (The Jackson Laboratory, Bar Harbor, ME, USA; B6.129S4 mixed background), and 7 adult (12–16 weeks old) VPA-exposed mice and 7 saline-exposed littermate mice (in-house breeding; CD1 background) and 173 adult (12–16 weeks old) male C57BL/6J mice (Charles River Laboratories, L’Arbresle, France). These 173 C57BL/6J mice were allocated among the reported tests as follows: 4 mice were used for establishment of dosing conditions for etomidate anesthesia. 32, 30 and 34 mice were employed in perfusion experiments under isoflurane, medetomidine and etomidate anesthesia, respectively, 26 mice were used in imaging genetics studies as age-matched controls for the BTBR T+tf/J mouse model of autism, and 47 C57BL/6J mice were used in pharmacological fMRI studies. All animals were group-housed (2–3 per cage) in a temperature-, humidity- and light-controlled environment (22–24 °C, 40–60%, 12 h light/dark cycle) and had access to food and water *ad libitum*. At the time of experiment, body weights were 250–300 g for SD rats and, depending on the strain, 20–50 g for mice.

### Establishment of dosing conditions for etomidate anesthesia

While animals were in a tubular restrainer, the lateral tail vein was cannulated with either a hypodermic or a butterfly needle which was connected via a catheter to an infusion pump (Harvard Apparatus, Boston, MA, USA). Anesthesia was induced with isoflurane (rats: 4%; mice: 2.5–3%; Abbot, Baar, Switzerland) delivered by a calibrated vaporizer in a carrier gas consisting of oxygen and air (1:5 v/v). Subsequently, etomidate (Etomidate-®Lipuro; B. Braun Melsungen AG, Melsungen, Germany) was administered as a primed continuous intravenous infusion. Isoflurane supply was discontinued after 8 min whereas the supply of oxygen-enriched breathing gas was maintained throughout the duration of the anesthesia. Species- and strain-specific dose-finding for etomidate anesthesia was carried out. The priming dose of etomidate was set to a rate of 4 mg/kg/min (mice) or 5 mg/kg/min (rats) for the initial 3 min. Thereafter, anesthesia was sustained with rates of 0.17, 0.25, 0.33 or 0.50 mg/kg/min for rats and 0.5 or 0.75 mg/kg/min for C57BL/6J mice, 0.75 or 1.0 mg/kg/min for BTBR T+tf/J mice or 0.75, 1.0, 1.2 or 1.5 mg/kg/min for CD1 mice (each n = 2 per dose). Anesthesia was continued for 2 h to reflect the maximum duration of a typical MRI assessment.

Body temperature was continuously monitored with a rectal probe and maintained at 37.3 ± 0.3 °C with a feedback-regulated electric heating blanket (Prang + Partner AG, Pfungen, Switzerland). Induction of anesthesia was assessed by the loss of the righting reflex^42^. The depth of anesthesia was further characterized by standardized tests for nociceptive (tail pinch), withdrawal (toe, ear pinch) or palpebral (medial eye canthus) reflexes and the response to vibrissal (mechanical deflection) or loud sound stimuli (hand clapping) carried out every 10 min. In addition, the color of the mucous membranes was evaluated. After discontinuation of etomidate infusion, animals were further monitored until they were fully ambulatory. Table 1 lists the species- and strain-specific infusion rates of etomidate as well as doses of other anesthetics tested in this study that produced adequate anesthetic depth and good recovery.

### Animal preparation, anesthesia, and monitoring for fMRI

On the day of the fMRI experiment, mice and rats were anesthetized either with isoflurane, medetomidine (Domitor®; Pfizer, Karlsruhe, Germany), or etomidate according to the conditions outlined in Table 1. Isoflurane was supplied with a calibrated vaporizer in a carrier gas composed of oxygen and air (1:5 v/v) to spontaneously breathing animals initially in an induction chamber and later via a face mask. Isoflurane concentration was adjusted such as to keep the depth of anesthesia constant, as indicated by a constant breathing rate of 50–60 breaths/min. Medetomidine was administered subcutaneously as a bolus-primed continuous infusion to animals initially anesthetized with isoflurane (see above) for the first 8 min. The bolus was injected manually with a syringe and hypodermic needle into the flank of the animal. For the infusion, a hypodermic needle connected via a catheter to an infusion pump was placed in the contralateral flank. After fMRI, medetomidine was antagonized by a subcutaneous injection of atipamezole (Antisedan; Pfizer, Karlsruhe, Germany; rats: 0.2 mg/kg; mice: 0.8 mg/kg). Etomidate anesthesia was carried out as established above, starting with an initiation phase supported by isoflurane followed by a primed continuous intravenous infusion of etomidate. All animals were breathing spontaneously except mice subjected to electrical stimulation paradigms where neuromuscular blockade was achieved with pancuronium bromide (Sigma-Aldrich, Steinheim, Germany; 1 mg/kg intravenous bolus). Thence, mechanical ventilation with a small animal ventilator (CWE Inc., Ardmore, PA, USA) was used to supply breathing gas at a rate of 1.8 ml/min over 80 breaths/min with a 1:3 ratio of inspiration and expiration.

When anesthetized, animals were prone positioned in a custom-built fiberglass cradle in the MRI scanner with the head immobilized in a stereotaxic holder by means of ear pieces and a bite bar. Ophthalmic ointment was applied to the eyes to prevent corneal drying. Respiratory rate, rectal body temperature, and oxygen and CO_2_ levels in the inspired and exhaled air were continuously monitored as previously described^43^. Body temperature was maintained at 37.3 °C with a feedback-regulated electric heating blanket.

After imaging, anesthesia was ceased and animals were kept on a heating pad until they showed spontaneous movements. Once fully ambulatory they were transferred back into their home cages. Suitability of etomidate for repeated fMRI was further assessed in a subset of mice (C57BL/6J: n = 10; BTBR T+tf/J: n = 6; CD1: n = 4) and rats (SD: n = 4). All 24 animals were imaged twice with a minimum intersession interval of one week and furthermore, all the mice were subjected to a third fMRI session after another week of rest.

### Functional stimulation paradigms

#### Pharmacological challenge

Cerebrovascular reserve capacity and hemodynamic responses to modulation of neural activity were investigated with two marketed drugs, i.e., acetazolamide (Diamox®; Vifor Ltd., Villars-sur-Glâne, Switzerland) and olanzapine (Toronto Research Chemicals Inc., North York, ON, Canada). Studies were carried out in a vehicle-controlled (saline or 0.3% Tween80 in 0.9% NaCl, respectively) parallel design with compound-treated and vehicle-treated animals being measured temporally interleaved. Groups were matched for controlled variables including strain, gender, age, weight, housing, feeding, handling and drug administration route. Acetazolamide was tested in rats and mice, olanzapine solely in C57BL/6J mice (Table 1).

Acetazolamide was injected under anesthesia after acquisition of three initial CASL volumes of baseline perfusion. A bolus of 30 mg/kg was administered either intraperitoneally in etomidate anesthetized animals or intravenously in isoflurane- and medetomidine-anesthetized animals, depending on the primary use of the tail vein catheter. Based on previous experience in our lab^27^,^43^, the inter-individual variability (standard deviation) of perfusion in an average-sized brain area was assumed to be around 10 ml/100 g/min, while we expected differences among anesthetics and effects of acetazolamide of 50 ml/100 g/min or more. Hence, with an expected effect size of >5, only very few animals per sample were sufficient to substantiate our hypothesis.

Olanzapine was administered intraperitoneally at a dose of 3 mg/kg 30 min prior to induction of anesthesia in order to allow pharmacological fMRI (phMRI) to be performed at the anticipated time of maximal drug exposure. Previous experience revealed an inter-individual variability of 7% in phMRI under isoflurane, whereas we consider pharmacology-related modulations interesting at 10% and above. Hence, a sample size of 9 per group was foreseen to target significance and power levels of 0.05 and 0.8, respectively, for an ordinary two-sample t-test. In total, 7 out of 54 animals either did not enter the MRI measurement due to difficulties in tail vein cannulation, or had to be discarded due to unsatisfactory image registration.

#### Peripheral electrical stimulation

In accord with previous studies performed in rodents^13^,^14^,^15^, mice were paralyzed using a muscle relaxant and mechanically ventilated to avoid motion artefacts potentially caused by the stimulation paradigm. For peripheral electrical stimulation, a pair of bipolar platinum needle electrodes (Genuine Grass Instruments, Warwick, RI, USA) was placed subcutaneously into each mouse hindpaw and was connected to a current stimulus isolator (A365D; World Precision Instruments Inc., Sarasota, FL, USA). Stimuli delivery was controlled with custom-written LabVIEW software (National Instruments, Austin, TX, USA) and synchronized to the onset of the fMRI acquisition. A fixed set of stimulation parameters (current amplitude = 2 mA, pulse duration = 0.5 ms, pulse frequency = 5 Hz) was used in all experiments. The stimulation paradigm consisted of a block design starting with a resting period of 120 s (baseline), followed by four cycles of a 20 s stimulus period and a 60 s post-stimulus period each. Each paw was stimulated twice in alternate order. The time between induction of anesthesia and the fMRI recording was kept constant at 30 min to ensure the same anesthesia and physiological conditions for all animals.

### fMRI acquisition and data analysis

#### Perfusion-based fMRI

Perfusion-based fMRI as a proxy for neural activity and neurovascular response was carried out on a Bruker Biospec 47/40 (rat studies) and Bruker Biospec 94/20 (mouse studies) MR systems (Bruker Biospin, Ettlingen, Germany). A volume resonator was used for signal excitation and an actively decoupled, quadrature surface coil was positioned over the head of the animal for signal reception. Following localization of the most rostral extension of the corpus callosum as a landmark on scout images, eight coronal image planes were selected at −10.0, −7.8, −5.3, −2.9, −1.6, −0.3, +1.0 and +2.3 mm from bregma^44^ for studies in rats and at −3.5, −2.6, −1.7, −0.8, +0.1, +1.0, +1.9 and +2.8 mm for mice^45^. All subsequent images were acquired in these planes, with a field of view of 40 × 40 mm^2^ and a slice thickness of 1.1 mm in rats, and 20 × 20 mm^2^ and 0.6 mm in mice, respectively. The first volume was a set of T_2_-weighted anatomical RARE images (rats: TR/TE_eff_ = 1,780/39 ms, RARE factor 8, matrix 256 × 256; mice: TR/TE_eff_ = 3,150/34 ms, RARE factor 8, matrix 256 × 256). Next, a T_1_ image series required to quantitate perfusion was obtained using an inversion-recovery snapshot FLASH sequence with eight inversion times (rats: TR/TE = 3,375/1.4 ms, TI = 89, 241, 393, 545, 698, 850, 1002 and 1154 ms, matrix 128 × 64; mice: TR/TE = 4,000/1.6 ms, TI = 96, 263, 429, 595, 761, 927, 1094 and 1260 ms, matrix 64 × 64)^46^. Finally, cerebral perfusion was assessed by continuous arterial spin-labelling (CASL)^47^ with single-slice centered-RARE readout (rats: TR/TE = 3,750 ms/5.7 ms, RARE factor = 32, matrix 128 × 64, labelling pulse 2.5 s, post labelling delay 0.4 s; mice: TR/TE = 3,750 ms/5.4 ms, RARE factor = 32, matrix 128 × 64, labelling pulse 3 s, post labelling delay 0.4 s). The total acquisition time for one volume of CASL images was 4 min. Depending on the experimental design, i.e., static or dynamic characterization, contiguous time series of either 3 or 12 volumes were acquired.

#### Blood oxygenation level dependent fMRI

Blood oxygenation level dependent (BOLD) fMRI was conducted on a Bruker Biospec 94/30 MR system (Bruker BioSpin, Ettlingen, Germany). A four–element receive–only cryogenic phased array coil (Bruker BioSpin, Ettlingen, Germany) was used in combination with a linearly polarized room temperature volume resonator for transmission. 12 contiguous coronal slices of 0.5 mm thickness were acquired with the first slice placed 2 mm rostral of bregma according to a stereotaxic mouse brain atlas^45^. All subsequent images were acquired in these planes, with a field of view of 16 × 7 mm^2^. An anatomical reference scan was acquired using a T_2_ weighted RARE sequence (TR/TE_eff_ = 4,000/39 ms, RARE factor 8, matrix 160 × 70). Local field homogeneity was optimized in the area of interest using previously acquired field maps. BOLD fMRI was performed using a gradient-echo echo-planar imaging (GE-EPI) sequence (TR/TE = 1,000/12 ms, flip angle 60°, matrix 80 × 35, 1 average) with a temporal resolution of 1 s and interleaved acquisition of slices. During the electrical stimulation paradigm, 440 repetitions were acquired.

#### Analysis of perfusion fMRI data

Both acquisition and analysis of images were performed in a highly automated way as further detailed below, thus excluding user bias. CASL images were processed and analyzed using in-house software written in IDL 6.4 (Interactive Data Language; Exelis, Boulder, CO, USA) and MATLAB 7.14 (The MathWorks Inc., Natick, MA, USA). The anatomical volume of each individual animal was co-registered to an in-house mouse- or rat-brain template using the open-source software SPM5 (Welcome Trust Centre for Neuroimaging, London, UK). Spatial normalization comprised a 12-parameter affine as well as a nonlinear transform, allowing for global scaling as well as for local adjustments of anatomical features. This normalization procedure was applied identically to all functional images of the same subject. The template was in alignment with an in-house generated digital atlas delineating selected anatomical brain areas adapted from standard brain atlases^44^,^45^. T_1_ maps per animal were calculated on a voxel-wise basis by fitting a 3-parameter exponential to the intensities across the eight inversion times. These T_1_ maps were then combined with the pertinent CASL images to obtain quantitative absolute perfusion maps, as described elsewhere^43^. Mean cerebral perfusion for individual animals was determined in the entire brain and in the prefrontal cortex (PFC) as a representative brain area of major interest. In case of static characterization, values were averaged across the 3 successive CASL volumes.

In the olanzapine studies, perfusion maps of each individual were normalized slice-wise to the brain-mean value in order to elucidate drug-induced spatial activation patterns while accounting for possible systemic changes affecting global brain perfusion (e.g., direct vascular effects of the drug) and eliminating part of the inter-individual variability. Group-difference maps were then generated by subtracting the mean normalized-perfusion maps of vehicle-treated animals from those of olanzapine-treated animals.

For visualization of perfusion data, standard deviations are depicted for static experiments to depict the spread of individual animals, whereas 95% confidence intervals (assuming t-distributed estimates of the mean, to account for small sample sizes) are shown for dynamic experiments to easily identify significant deviations from baseline values. For static experiments, ordinary two-sample, two-tailed t-tests were performed for each species/strain and each brain area to confirm perfusion differences between anesthetics. For dynamic experiments, a mixed-effects model was specified for each species/strain and each brain area, with “anesthesia” and “time” as between- and within-subject factors, respectively. Comparison of CVRC between anesthetics was then performed using a post-hoc contrast jointly testing the 5 time points around the presumed peak response (i.e., 8 to 24 min after injection of acetazolamide).

#### Analysis of BOLD fMRI data

For analysis of BOLD fMRI data, spatial pre-processing and generation of statistical parametric maps (activity maps) were performed using a custom made script, based on AFNI (http://afni.nimh.nih.gov). Regions of interest were defined according to a stereotaxic mouse brain atlas^45^ for the contralateral primary somatosensory hindlimb cortex (S1HL), secondary somatosensory cortex (S2) and thalamus (Thal) using MATLAB 7.14. The initial 10 imaging volumes were discarded to allow magnetization to reach steady state. All images of a scan series were then slice-time corrected and co-registered to the same template. Prior to analysis, the time series data were low-pass filtered at a cutoff frequency of 0.32 Hz to diminish respiratory-induced noise, and de-trended using a first order polynomial fit on the pre-stimulus baseline to account for scanner drift. After smoothing with a 0.4 mm Gaussian kernel, statistical parametric maps were generated using the general linear model approach with a three-parameter basis function as hemodynamic response function. The obtained contrast images that should reflect stimulus-induced activations in each animal were entered into a second level random-effects analysis using a one-way ANOVA. The resulting F-statistics were corrected for multiple comparisons. Monte Carlo simulations based on the average estimated smoothness of the datasets were used to determine the minimum cluster size corresponding to a corrected *p* value of 0.05. Stimulus-evoked BOLD signal changes were expressed as percentages relative to baseline.

## Additional Information

**How to cite this article**: Petrinovic, M. M. *et al.* A novel anesthesia regime enables neurofunctional studies and imaging genetics across mouse strains. *Sci. Rep.* **6**, 24523; doi: 10.1038/srep24523 (2016).

## Supplementary Material

Supplementary Information

## Figures and Tables

**Table 1:** Summary of anesthesia and treatment protocols.

Anesthetic agent (application route)	Respiratory conditions	Animals		Initial (priming) dose	Maintenance dose	Challenge
Isoflurane (Inhalation)	Spontaneously breathing (carrier gas composed of oxygen and air, 1:5 v/v)	rat	Sprague Dawley	4%	2.0–2.3%	Acetazolamide (30 mg/kg)
		mouse	C57BL/6J	2–3%	1.8–2.0%	Acetazolamide (30 mg/kg) Olanzapine (3 mg/kg; C57BL/6J mice)
			BTBR T+tf/J			
			CD1			
Medetomidine^a^(s.c.)		rat	Sprague Dawley	0.2 mg/kg (+4% isoflurane)	0.1 mg/kg/h	Acetazolamide (30 mg/kg)
		mouse	C57BL/6J	0.3 mg/kg (+2.5–3% isoflurane)	0.6 mg/kg/h	Acetazolamide (30 mg/kg) Olanzapine (3 mg/kg)
Etomidate^b^(i.v.)		rat	Sprague Dawley	5 mg/kg/min (3 min) (+4% isoflurane)	0.25 mg/kg/min	Acetazolamide (30 mg/kg)
		mouse	C57BL/6J	4 mg/kg/min (3 min) (+2.5–3% isoflurane)	0.75 mg/kg/min	Acetazolamide (30 mg/kg) Olanzapine (3 mg/kg; C57BL/6J mice) Electrical hindpaw stimulation (2 mA; ventilated C57BL/6J mice)
			BTBR T+tf/J		1.0 mg/kg/min	
			CD1		1.5 mg/kg/min	

^a^i.v., intravenous.

^b^s.c., subcutaneous.
