# Supplementary material for: A novel anesthesia regime enables neurofunctional studies and imaging genetics across mouse strains
Source: Sci Rep. 2016 Apr 15;6:24523. doi: 10.1038/srep24523 (PMC4832200; doi:10.1038/srep24523)
Supplement: Supplementary Information [file srep24523-s1.pdf]

SUPPLEMENTARY INFORMATION

A novel anesthesia regime enables neurofunctional studies and imaging genetics across mouse strains

Marija M. Petrinovic[1], Georges Hankov[2], Aileen Schroeter[2,3], Andreas Bruns[1], Markus Rudin[2,3,4], Markus von Kienlin[1], Basil Künnecke[1], Thomas Mueggler[1*]

[1]Roche Pharmaceutical Research & Early Development, Neuroscience Discovery, Roche Innovation Center Basel, F. Hoffmann-La Roche Ltd, Grenzacherstrasse 124, 4070 Basel, Switzerland

[2]Institute for Biomedical Engineering, University and ETH Zurich, Wolfgang-Pauli-Strasse 27, 8093 Zurich, Switzerland

[3]Neuroscience Center Zurich, University and ETH Zurich, Winterthurerstrasse 190, 8057 Zurich, Switzerland

[4]Institute of Pharmacology and Toxicology, University of Zurich, Winterthurerstrasse 190, 8057 Zurich, Switzerland

Supplementary Figure S1

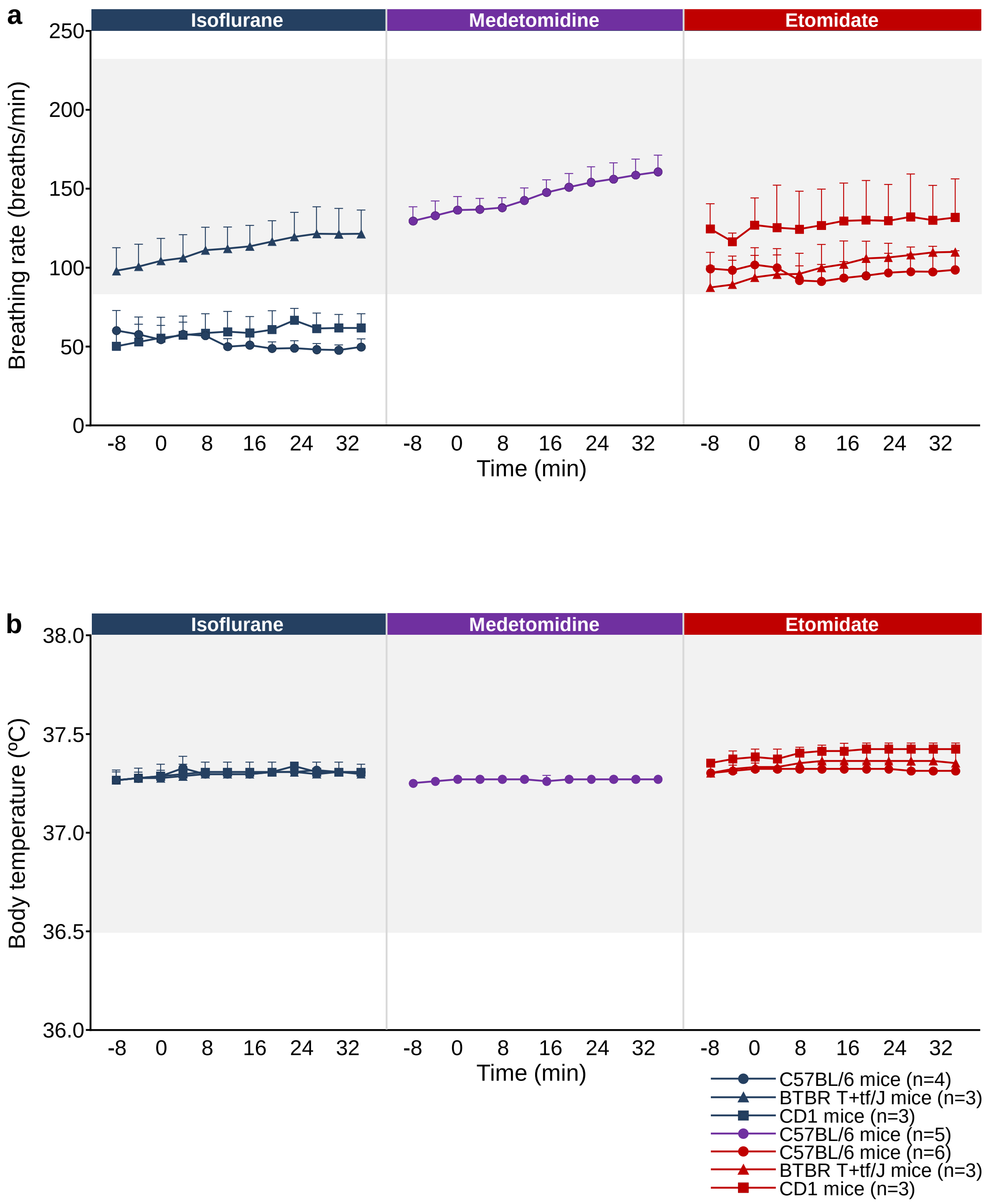

Supplementary Figure S1. Comparison of physiological parameters during the fMRI acquisitions at baseline conditions and during acetazolamide challenge under the different anesthesia regimens in C57BL/6J, BTBR T+tf/J and CD1 mice. (**a**) Breathing rate. (**b**) Body temperature. Acetazolamide was administered at t = 0. The gray-shaded areas designate the physiological spans. Line charts represent sample means; error bars represent standard deviations to visualize the spread of individual animals. Sample sizes are provided in each legend.

Supplementary Figure S2

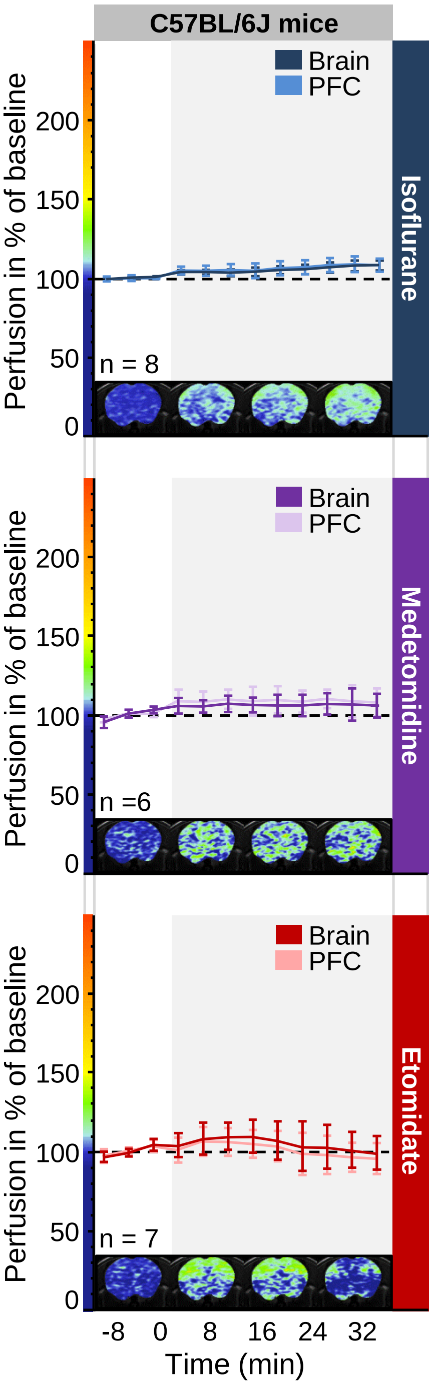

Supplementary Figure S2. Cerebral perfusion in freely breathing C57BL/6J mice upon intraperitoneal injection of saline (vehicle) as a negative control under different anesthetics. Graphs show the time course of the perfusion response in whole brain and the PFC before and after the injection of saline under (**a**) isoflurane, (**b**) medetomidine and (**c**) etomidate-based anesthesia. Saline was injected intravenously in isoflurane- and medetomidine-anesthetized animals and intraperitoneally under etomidate. Changes in absolute perfusion are presented as group-mean percent change relative to baseline (black dashed line at 100%) to allow comparison of different regions of interest. The left border of the gray-shaded area designates the time-point of saline application. Color-coded magnetic resonance images represent the percentage change of perfusion (according to the color bar along the y axis) as an average over three consecutive time-points for a representative coronal plane (+1.34 mm relative to bregma). Line charts represent sample means; error bars represent 95% confidence intervals to visualize significant deviations from baseline. Group sample sizes are provided below each graph. PFC, prefrontal cortex.
